# Supplementary material for: Seminoma and Embryonal Carcinoma Footprints Identified by Analysis of Integrated Genome-Wide Epigenetic and Expression Profiles of Germ Cell Cancer Cell Lines
Source: PLoS One. 2014 Jun 2;9(6):e98330. doi: 10.1371/journal.pone.0098330 (PMC4041891; doi:10.1371/journal.pone.0098330)
Supplement: File S1 — ZIP file containing DMRforPairs output for significant regions. Please start from the html files. (ZIP) [file pone.0098330.s008.zip › figures/18181.pdf]

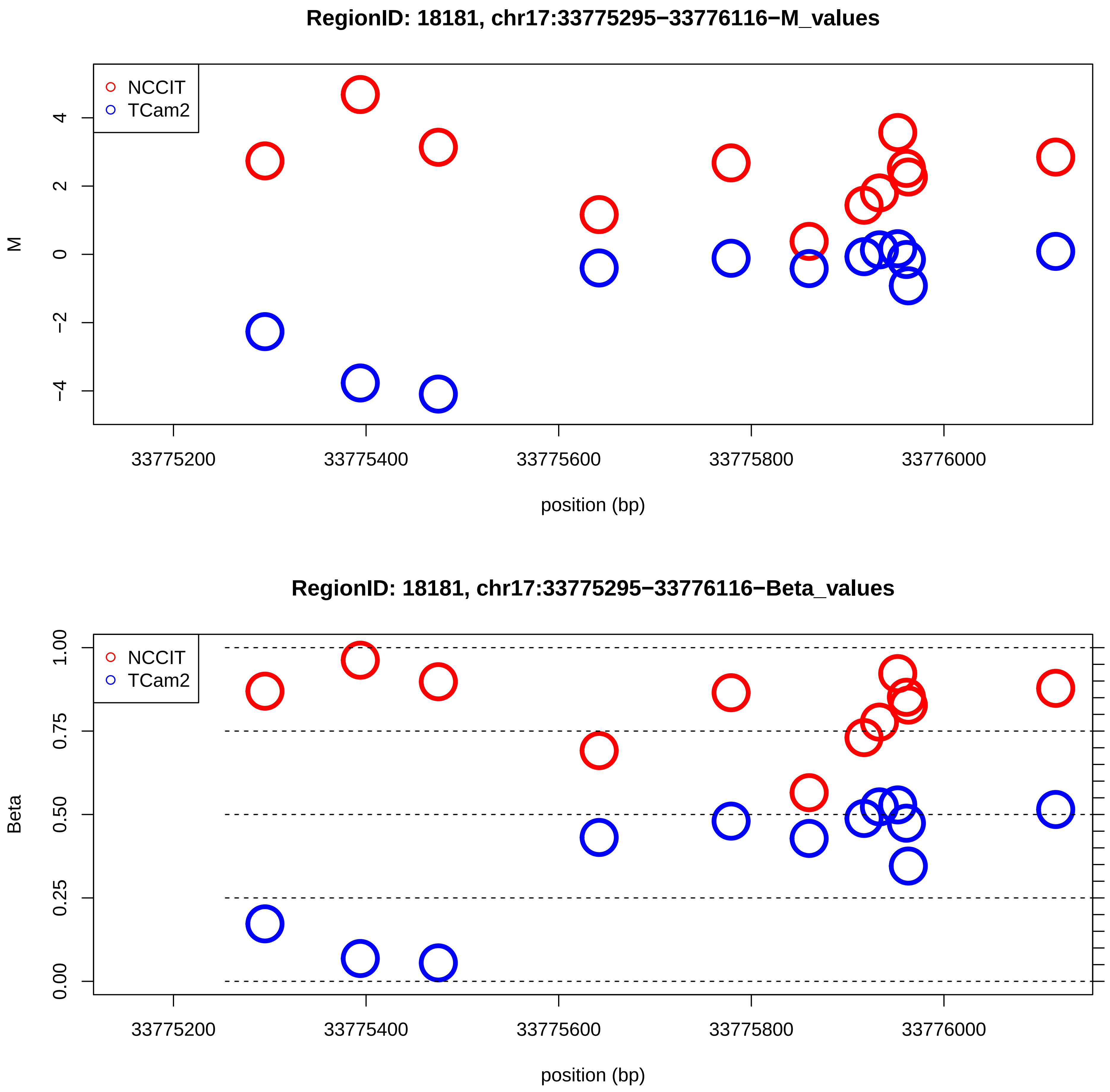
RegionID: 18181, chr17:33775295−33776116−M_values
NCCIT
TCam2
M
−4
−2
0
2
4
33775200
33775400
33775600
33775800
33776000
position (bp)
RegionID: 18181, chr17:33775295−33776116−Beta_values
NCCIT
TCam2
Beta
0.00
0.25
0.50
0.75
1.00
33775200
33775400
33775600
33775800
33776000
position (bp)

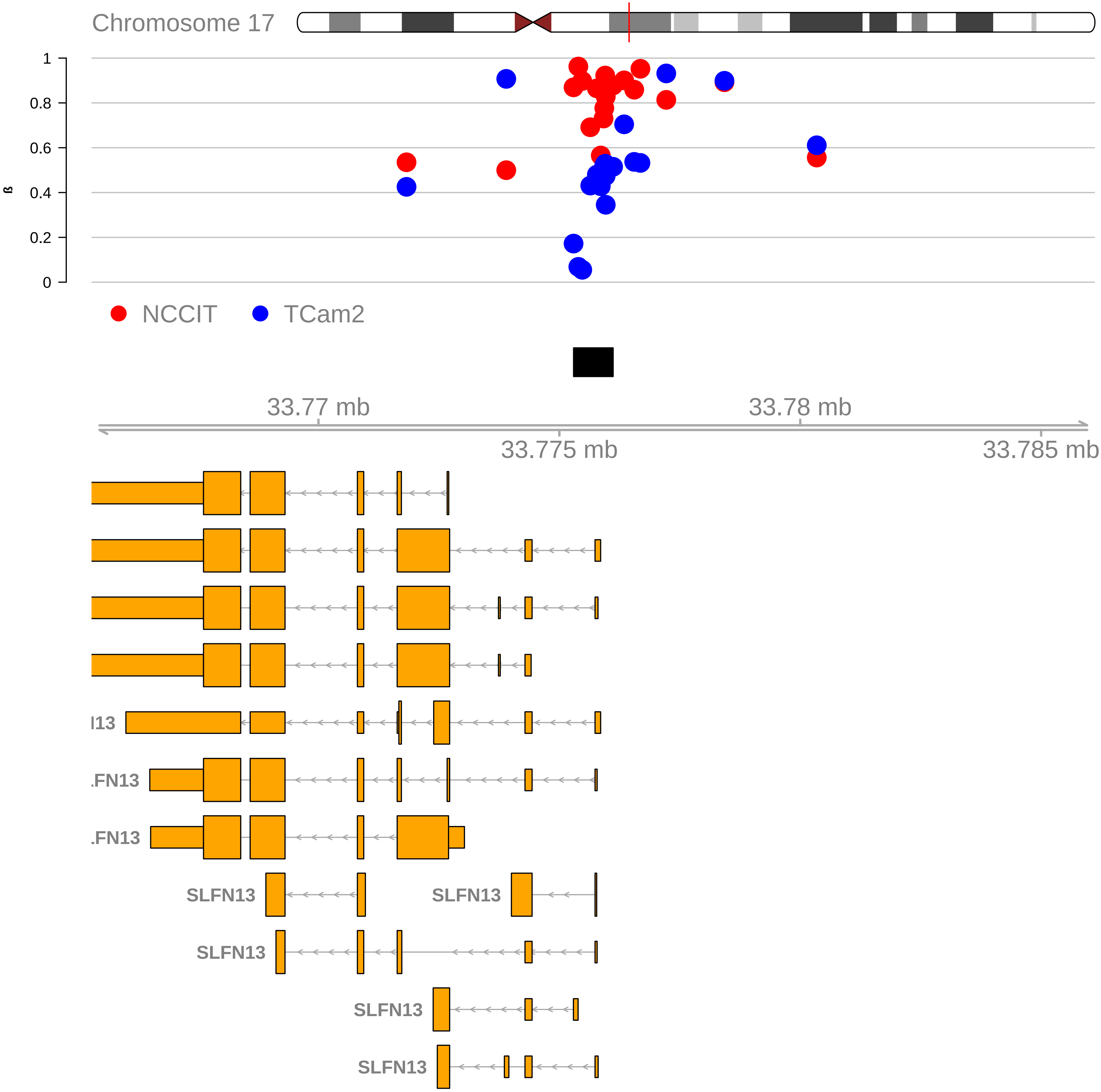

Chromosome 17
ß
1
0.8
0.6
0.4
0.2
0
NCCIT
TCam2
33.77 mb
33.78 mb
33.775 mb
33.785 mb
l13
.FN13
.FN13
SLFN13
SLFN13
SLFN13
SLFN13
SLFN13
